# A Literature Review of Mathematical Models of Hepatitis B Virus Transmission Applied to Immunization Strategies From 1994 to 2015

**DOI:** 10.2188/jea.JE20160203

**Published:** 2018-05-05

**Authors:** Peifeng Liang, Jian Zu, Guihua Zhuang

**Affiliations:** 1 Department of Statistics, People’s Hospital of Ningxia Hui Autonomous Region, Yinchuan, China; 2 Department of Applied Mathematics, School of Mathematics and Statistics, Xi’an Jiaotong University, Xi’an, China; 3 Department of Epidemiology and Biostatistics, School of Public Health, Xi’an Jiaotong University Health Science Center, Xi’an, China

**Keywords:** hepatitis B, transmission dynamic, compartmental model

## Abstract

A mathematical model of the transmission dynamics of infectious disease is an important theoretical epidemiology method, which has been used to simulate the prevalence of hepatitis B and evaluate different immunization strategies. However, differences lie in the mathematical processes of modeling HBV transmission in published studies, not only in the model structure, but also in the estimation of certain parameters. This review reveals that the dynamics model of HBV transmission only simulates the spread of HBV in the population from the macroscopic point of view and highlights several main shortcomings in the model structure and parameter estimation. First, age-dependence is the most important characteristic in the transmission of HBV, but an age-structure model and related age-dependent parameters were not adopted in some of the compartmental models describing HBV transmission. In addition, the numerical estimation of the force of HBV infection did not give sufficient weight to the age and time factors and is not suitable using the incidence data. Lastly, the current mathematical models did not well reflect the details of the factors of HBV transmission, such as migration from high or intermediate HBV endemic areas to low endemic areas and the kind of HBV genotype. All of these shortcomings may lead to unreliable results. When the mathematical model closely reflects the fact of hepatitis B spread, the results of the model fit will provide valuable information for controlling the transmission of hepatitis B.

## INTRODUCTION

Infection with hepatitis B virus (HBV) is a challenge to global health. There are more than 350 million chronic carriers of HBV and 0.6 million deaths per year due to HBV-related liver disease or hepatocellular carcinoma.^1^^–^^3^ To date, some comprehensive tactics to eliminate HBV transmission have been implemented with considerable success. Immunizing susceptible individuals, especially newborns, with safe and effective vaccines is the most attractive and most economical way to reduce the incidence of hepatitis B, in terms of both cost-effectiveness and cost-benefit ratios.^4^^–^^9^ Despite the success of immunization, challenges still remain. So, it is crucial to predict the long-term trends in HBV prevalence and provide useful information for public health decision-making. One feasible method to predict the prevalence of infectious disease is to use a mathematical model.

The transmission dynamics model, also known as the compartmental model, is an important theoretical epidemiology method used to study the transmission dynamics of infectious disease. The transmission dynamics models is based on the population characteristics, the infection characteristics of the infectious disease, and related social factors, and is used to analyze the dynamic behavior of infectious disease and to do some mathematical simulations. The resulting model is conducive to predicting the transmission tendency of the disease, determining key factors that influence the spread of disease, and seeking optimal strategies for disease control and prevention.^10^^,^^11^ Particularly, this method can allow researchers to add the indirect effects of herd immunity into vaccine effectiveness.^12^^,^^13^ In the early 1980s, the transmission dynamics model was first used to study the transmission dynamics of hepatitis B and the effectiveness of control. With the introduction of available hepatitis B vaccine, how to use a mathematical model to predict the long-term effects of vaccination on hepatitis B control became the main focus. McLean and Blumberg first proposed a differential equation model of HBV transmission to address questions concerning the impact of a mass vaccination program on the prevalence status of hepatitis B in 1994.^14^ Since then, many researchers have studied the transmission dynamics of HBV and assessed the effects of different vaccine strategies using similar methods to McLean and Blumberg. Well-supported results of such research have given a quantitative basis for making optimal decisions in public health policy regarding HBV transmission. There are, however, differences in the mathematical modeling process of HBV transmission in published studies, not only in the model structure, but also in the estimation of certain parameters. All of these factors are the important determinants of outcomes and can significantly influence the final results.

This study conducted a literature review of the existing research on mathematical models of hepatitis B transmission under different vaccine strategies. The main aims of this study are: (i) to describe the methodological characteristics of existing studies and evaluate their strengths and limitations, and: (ii) to summarize the main results of existing studies regarding the impact of different vaccination strategies. This study is intended to offer valuable insight into the compartmental models of HBV transmission.

## METHODS

### Literature search strategy

We conducted a literature search for relevant articles, written in English, using PubMed, OVID, SODL, and Web of science. Web of science is a comprehensive retrieval platform with access to eleven electronic databases, including Medline. The search was confined to articles published from January of 1994 through December of 2015. To minimize the chance of missing an important study, we also performed a manual search of the references of all articles found in our search, including any potentially eligible studies that were found using Google Scholar. The research MeSH, or keywords, were defined as: (“hepatitis B” OR “HBV”) AND (“modeling” OR “mathematics model”) AND (“vaccine” OR “vaccination” OR “immunization.”).

### Inclusion criteria

In this study, the following inclusion criteria were used to determine whether a study was eligible:

(a) Research objectives: the study was used to evaluate the potential impact of vaccination strategies on the HBV transmission on a population-based level.
(b) Study method: the study was developed using a compartmental model.
(c) Language: the study was published in English.
(d) Text availability: the study was available in full text.
(e) Redundancy: the study was the most recent in a series of articles with the same first authors and similar modeling structure and content.

### Records screening and data extraction

Titles and abstracts of articles identified using the previously described search strategy were imported into Endnote X6 bibliographic software (Clarivate Analytics, Philadelphia, PA, USA), in which duplications of articles were removed. Two independent reviewers systematically screened the compiled records for potentially eligible studies using the inclusion criteria. Any disagreement between the two reviewers was resolved via discussion in order to come to a consensus. The key characteristics of the final articles selected were extracted using a pre-designed data extraction scheme. The scheme included the model structure used in the study, the basic parameters used in the model, and the outcome of the study. All data extraction was performed by the first author of this study and cross-checked by another researcher.

## RESULTS

### Characteristics of included studies

**Figure 1.**
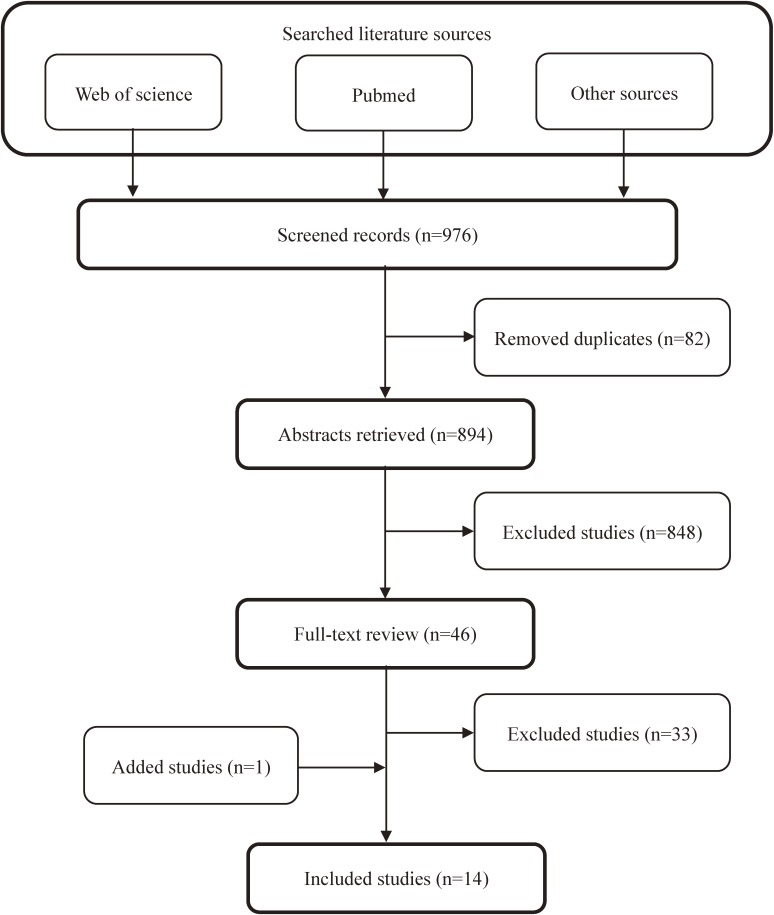
Flowchart of the search process.

Based on the literature search strategy, 14 relevant studies were identified as eligible studies and included in the review. Figure 1 shows the flowchart of the literature search process. Through abstract and full-text review, we excluded 881 studies that were not qualified due to one of the following reasons: (i) the study objective was not to assess the potential impact of vaccination strategies on the HBV transmission on a population-based level (*n* = 837); (ii) the model used in the study was not a compartmental model in structure (*n* = 37); (iii) the study was not available in full-text format (*n* = 4); (iv) the study was not published in English (*n* = 2); and (v) the study contained the same first author and a similar model structure as another study that had already been included (*n* = 1). Among the 14 included studies, 8 were done using a population with high HBV prevalence,^15^^–^^22^ one was done using a population with intermediate HBV prevalence,^23^ three were done using a population with low HBV prevalence,^24^^–^^26^ and one was done using a population where there was both high and low HBV prevalence.^27^ One included study normalized the population size to 1 but did not specify the endemic state of the population studied.^28^

### Model structure

#### Epidemiological compartments

**Figure 2.**
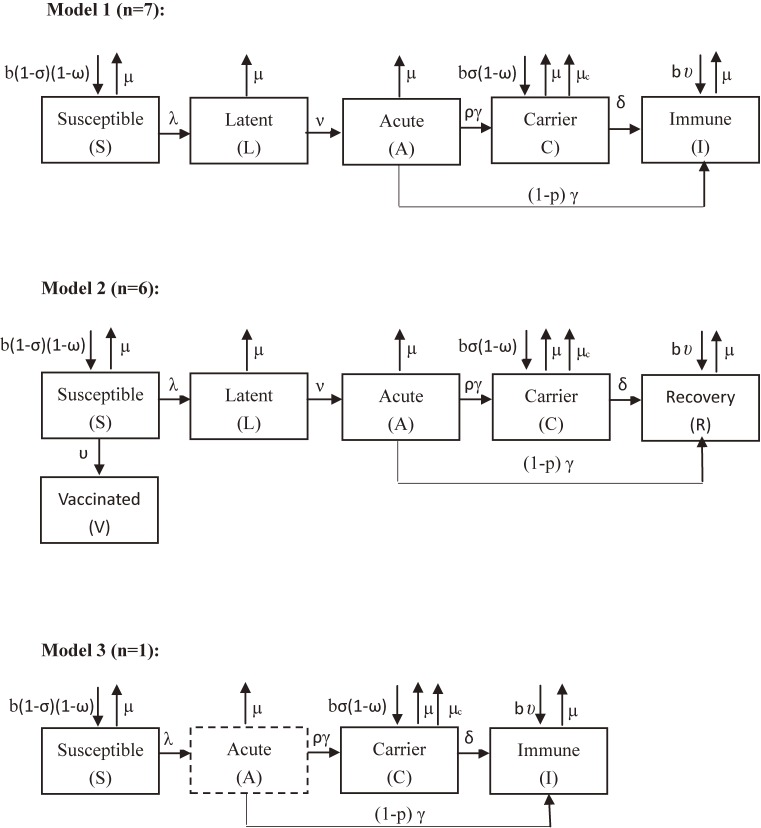
The epidemiological compartments and definitions. Susceptible (S), at risk of infection with HBV; Latent (L), individuals who have been infected but are not yet infectious; Acute (A), Individuals who are in the initial highly infectious stage of HBV infection; Carrier (C), people with chronic HBV infection who are infectious or non-infectious to others; Immune (I), individuals who have recovered from the carrier stage or acute stages of HBV infection or have been successfully immunized; Recovery (R), individuals who have recovered from the carrier stage or acute stages of HBV infection; Vaccinated (V), individuals who have been successfully immunized; “λ”, the force of HBV infection; “ν”, the rate at which individuals leave the latent class, “γ”, the rates at which individuals leave the acute class; “δ”, the recovery rate of carriers; “ρ”, the probability for an individual suffering from acute HBV infection to become a chronic carrier; “σ”, the proportion of perinatally infected, “ω”, proportion of births with successful vaccination, “υ”, the rate of successful vaccination, “φ”, the rate of waning vaccine-induced immunity; “b”, the birth rate, “μ”, the natural mortality rate, “μ_c_”, the HBV related mortality rate.

Compartmentalizing the epidemiological groups in terms of an individual’s state of HBV infection is the basis for constructing mathematical models. Five or six compartmental model is the most common structure in modeling the transmission dynamics of Hepatitis B, which was developed in 9 or 4 recruited eligible studies, respectively. As shown in Figure 2, for the five-compartment model (Model 1), the total population was divided into “Susceptible (S)”, “Latent (L)”, “Acute (A)”, “Carrier” (C), and “Immune (I)” according to differences in the epidemiological status of their HBV infection. “Susceptible (S)” denotes individuals at risk of infection with HBV; “Latent (L)” denotes individuals who have been infected but are not yet infectious; “Acute (A)” denotes individuals who are in the initial highly infectious stage of HBV infection; “Carrier (C)” denotes people with chronic HBV infection who are infectious or non-infectious to others; and “Immune (I)” denotes individuals who have recovered from the carrier stage or have been successfully immunized by vaccine. For the six-compartment model (model 2), the populations were categorized like the five-compartment model except that “Immune (I)” was further partitioned into “Recovery (R)” and “Vaccinated (V)”. This method took into account the fact that immunity after recovery lasts for the lifetime of the individual, while the immunity that follows vaccination may wane over time. Model 3 developed the three-compartment model: “susceptible”, “immune or vaccination”, and “chronic infection”; the “acute infection” was not considered as a compartment of the model but as a transient process by which a susceptible person would obtain immunity, become chronically infected or die due to fulminant hepatitis.

#### Mathematical expression of compartmental model

Ordinary differential equation is the most used mathematical expression in modeling the transmission dynamics of Hepatitis B, which was developed in 7 studies.^17^^–^^19^^,^^21^^,^^23^^,^^27^^,^^28^ In model 2, for example, transmission dynamics are modeled using six ordinary differential equations (Figure 2):

$$\{\begin{array}{l} \frac{dS}{dt}=b(1-\sigma )(1-\omega C)+\varphi V-(\mu +\lambda +\upsilon )S,\\ \frac{dL}{dt}=\lambda S-(\mu +\nu )L,\\ \frac{dI}{dt}=\nu L-(\mu +\gamma )I,\\ \frac{dC}{dt}=b\sigma (1-\omega )C+\rho \gamma I-(\mu +\mu_{c}+\delta )C,\\ \frac{dR}{dt}=(1-\rho )\gamma I+\delta C-\mu R,\\ \frac{dV}{dt}=b\omega +\upsilon S-(\varphi +\mu )V, \end{array}$$ (1)

where S, L, I, C, R, and V denotes the proportion of individuals at the stage of susceptible, latent, acute, carrier, recovery, and vaccinated to HBV in the total population, respectively. *t* is time, λ is the force of HBV infection, σ is the proportion of perinatal infection, ν is the rate at which individuals leave the latent class, γ is the rates at which individuals leave the acute class, δ is the recovery rate of carriers, ρ is the probability for an individual suffering from acute HBV infection to become a chronic carrier, υ is the rate of successful vaccination, ω is proportion of births with successful vaccination, φ is the rate of waning vaccine-induced immunity, b is the birth rate, μ is the natural mortality rate, μ_c_ is the HBV related mortality rate. In these models, all of the parameters are assumed to be constant.

Considering that age is one of the most important characteristics in the modeling of populations and infectious diseases. Some researchers^15^^,^^16^^,^^20^^,^^24^^,^^25^ developed age-dependent mathematical models for studying the transmission dynamics of Hepatitis B. Zhao et al^16^ considered the following partial differential equation model with age structure:

$$\{\begin{array}{l} \frac{\partial S(a,t)}{\partial a}+\frac{\partial S(a,t)}{\partial t}=-[\lambda (a,t)+\upsilon (a,t)+\mu (a)]S(a,t),\\ \frac{\partial L(a,t)}{\partial a}+\frac{\partial L(a,t)}{\partial t}=\lambda (a,t)S(a,t)-[\nu +\mu (a)]L(a,t),\\ \frac{\partial I(a,t)}{\partial a}+\frac{\partial I(a,t)}{\partial t}=\nu L(a,t)-[p(a)+\gamma +\mu (a)]I(a,t),\\ \frac{\partial C(a,t)}{\partial a}+\frac{\partial C(a,t)}{\partial t}\\ \;=p(a)I(a,t)-[\delta (a)+\mu (a)+\mu_{c}(a)]C(a,t),\\ \frac{\partial R(a,t)}{\partial a}+\frac{\partial R(a,t)}{\partial t}\\ \;=\upsilon (a,t)S(a,t)+\gamma I(a,t)+\delta (a)C(a,t)-\mu (a)R(a,t), \end{array}$$ (2)

where S, L, I, C, and R denote the proportion of individuals at the stage of susceptible, latent, acute, carrier, and immune to HBV in the total population, respectively. *a* is age and *t* is time. The force of infection (λ), the rate of successful vaccination (υ) are age- and time-dependent; the natural mortality rate (μ) and HBV related mortality rate (μ_c_) are age-dependent. The proportion of perinatal infection (ω), the rate at which individuals leave the latent class (ν), the rates at which individuals leave the acute class (γ), the recovery rate of carriers (δ), and the probability for an individual suffering from acute HBV infection to become a chronic carrier (p) are assumed to be constant.

Particularly, Mann et al^26^ discretized the population into five age classes. This study then constructed a difference equation model that includes the ages of the individuals experiencing a specific HBV clinical outcome. The mathematical model is given as:

For group 1 (0–1.25 years old),

$$\{\begin{array}{l} S_{1}=\left(\varepsilon_{0}(t)-\varepsilon_{1}(t)\omega {\frac{C_{4}}{N_{4}}}_{1}\right)b(t)-(\lambda_{1}+\mu_{1})S_{1},\\ L_{1}=\lambda_{1}S_{1}-(\nu_{1}+\mu_{1})L_{1},\\ I_{1}=\nu_{1}L_{1}-(\gamma_{1}+\mu_{1})I_{1},\\ C_{1}=\varepsilon_{1}\omega \frac{C_{4}}{N_{4}}b(t)+\rho_{1}\gamma_{1}I_{1}-(\delta +\mu_{1})C_{1},\\ R_{1}=(1-\omega_{0}(t))b(t)+(1-\rho_{1})\gamma_{1}I_{1}+\delta C_{1}-\mu_{1}R_{1}. \end{array}$$ (3)

For groups 2–5 (1.25–6 years old; 6–16 years old; 16–45 years old, and 45–70 years old),

$$\{\begin{array}{l} S_{j}=(1-\upsilon_{j-1}(t))\mu_{j-1}S_{j-1}-(\lambda_{j}+\mu_{j})S_{j},\\ L_{j}=\mu_{j-1}L_{j-1}+\lambda_{j}S_{j}-(\nu_{j}+\mu_{j})L_{j},\\ I_{j}=\mu_{j-1}I_{j-1}+\nu_{j}L_{j}-(\gamma_{j}+\mu_{j})I_{j},\\ C_{j}=\mu_{j-1}C_{j-1}+\rho_{j}\gamma_{j}I_{j}-(\delta +\mu_{j})C_{j},\\ R_{j}=\mu_{j-1}R_{j-1}+\upsilon_{j-1}(t)\mu_{j-1}S_{j-1}+(1-\rho_{j})\gamma_{j}I_{j}+\delta C_{j}-\mu_{j}R_{j}, \end{array}$$ (4)

where S, L, I, C, and R denote the proportion of individuals at the stage of susceptible, latent, acute, carrier, and immune to HBV in the total population, respectively. *j* is the age group, *t* is time. *b* is the birth rate, ε_0_ is the proportion of unvaccinated babies who born to non-carrier mothers in all babies, ε_1_ is the proportion of babies born to carrier mothers in all babies. In this model, the recovery rate of carriers (δ) are assumed to be constant; the force of infection (λ), the rate of successful vaccination (υ), the natural mortality rate (μ), the proportion of intrauterine infected (ω), the rate at which individuals leave the latent class (ν), the rates at which individuals leave the acute class (γ), the recovery rate of carriers (δ), and the probability for an individual suffering from acute HBV infection to become a chronic carrier (ρ) are age-dependent.

#### Population dynamics factors

For population dynamics factors, 7 studies^17^^,^^18^^,^^21^^,^^23^^,^^25^^,^^27^^,^^28^ assume population size keeps constant in a closed environment, in which the birth and death rates of a population are equal during the epidemic period of hepatitis B, and that there is no HBV related death. Six studies^15^^,^^16^^,^^19^^,^^20^^,^^24^^,^^26^ considered that the birth and death rates of a population are differ as the population size varies, which means that there is a migration from high or intermediate HBV endemic areas to low endemic areas, or there is a HBV-related death case.

### Parameter estimation

Parameter estimation is one of the most important works involved in the modeling process. The key parameter values reviewed in the 14 papers are summarized in Table 1.

**Table 1.** The key parameters used in numerical simulation for the models

Authors (year)	Force of infection (λ)	Proportion of acute infection individuals become chronic carrier (ρ)	Rate at which individuals leave the latent class (ν)	Rate at which individuals leave the acute class (γ)	Recovery rate of carriers (δ)	Proportion of perinatal infection (acute or carrier mothers)
Edmunds, et al (1996)^15^	Age-time-dependent, estimated from serological data using polynomial catalytic infection model λ: 0.159 (0–1 years), 0.144 (1–5 years), 0.116 (5–10 years), 0.089 (10–15 years), 0.030 (15–80 years) $\lambda_{\text{g}}(\text{a},\text{t})=\{\begin{array}{l} \pi (\text{a},\text{t}),\text{a}<\text{15 years}\\ \pi (\text{a},\text{t})+\rho_{\text{g}}(\text{t}),\text{a}\geq \text{15 years} \end{array}$ π(a, t): horizontal transmission, ρ_g_(t): Sexual transmission $\pi_{\text{i}}(\text{t)}=\frac{\sum_{\text{j}=1}^{\text{n}}\beta_{\text{ij}}\{{\bar{\text{Y}}}_{\text{jm}}(\text{t})+{\bar{\text{Y}}}_{\text{jf}}(\text{t})+\alpha [{\bar{\text{C}}}_{\text{jm}}(\text{t})+{\bar{\text{C}}}_{\text{jf}}(\text{t})]\}}{\sum_{\text{j}=1}^{\text{n}}\left\{{\bar{\text{N}}}_{\text{jm}}(\text{t})+{\bar{\text{N}}}_{\text{jf}}(\text{t})\right\}}$ β_ij_ denotes transmission coefficient and calculated from force of infection.	$\text{p(a)}=\{\begin{array}{l} 0.89,\text{a}\leq 0.5\text{ year}\\ \text{exp}(-0.65a^{0.46}),\text{a}>0.5\text{ years} \end{array}$	6 per year	4 per year	0.025 per year	Acute: 0.711; Carrier: 0.109
Williams, et al (1996)^24^	Age-time-dependent. Sexual transmission: $\lambda_{\text{gs}}(\text{a},\text{t})={\text{c}}_{\text{gs}}(\text{a})\frac{\sum_{\text{r}}\int_{\alpha_{1}}^{\alpha_{2}}{\text{c}}_{\text{hr}}(\alpha )[\beta_{1}{\text{Y}}_{\text{hr}}(\text{t},\text{a})+\beta_{2}{\text{C}}_{\text{hr}}(\text{t},\text{a})]\text{d}\alpha }{\sum_{\text{r}}\int_{\alpha_{1}}^{\alpha_{2}}{\text{c}}_{\text{hr}}(\alpha ){\text{N}}_{\text{hr}}(\text{t},\alpha )\text{d}\alpha }$ g and h representing opposite sexes, α_1_ and α_2_ the ages of starting and ceasing sexual activity, β_1_ and β_2_ the probability of transmission from partnership with acutely and carrier individual. α_1_ and α_2_ were estimated from National survey data; β_1_ and β_2_ based on the previous paper. β_1_: heterosexual: 0.33, homosexuals: 0.46 β_2_: heterosexual: 0.25, homosexuals: 0.30	Infant: 0.885; Adult: 0.1	8.677 per year	3.467 per year	0.015 per year	Acute: 0.724; Carrier: 0.115
Zhao, et al (2000)^16^	Age-time-dependent, estimated from serological data using polynomial catalytic infection model $\lambda (\text{a},0)=\{\begin{array}{l} 0.13074116-0.01362531\text{a}+0.00046463{\text{a}}^{2}\\ -0.00000489{\text{a}}^{3},0\leq \text{a}\leq 47.5\text{ year}\\ \lambda (47.5,0),\text{ a}>47.5\text{ years} \end{array}$ $\lambda (a,t)=\int_{0}^{E}\beta (a^{\prime },a)[T(a^{\prime },t)+C(a^{\prime },t)]da^{\prime }$	p(a) = 0.706004 exp(0.787711a) + 0.084648	8 per year	4 per year	0.01 (5–45 years), 0.045 (50–60 years), 0.08 (65 years)	Ignore intrauterine infection, the intrapartum and postpartum infection were reflected in the force of HBV infection
Medley, et al (2001)^27^	Time-dependent, estimated from a set of data of previous study. λ is related with the average age at infection, the higher the average age at infection, the lower the rate of infection (λ). λ = β(y + αc), where α is the infectiousness of carriers relative to acute infections, and β is the transmission coefficient, representing the rate of the contacts and probability of transmission between individuals within the population.	p(λ) = f + (1 − f)exp[0.645λ^−0.455^] f is the lowest value that this probability can take, and is essentially the probability of carriage development in adults.	6 per year	4 per year	0.025 per year	Acute: ignored Carrier: 0.11
Thornley, et al (2008)^17^	Time-dependent, λ = β(y + αc)	p(λ) = f + (1 − f)exp[−0.645λ^−0.455^]	6 per year	4 per year	0.025 per year	Carrier: 0.11
Kretzschmar, et al (2009)^25^	Age-time-dependent. Sexual transmission: following Williams, et al (1996) $\lambda_{\text{gs}}(\text{a},\text{t})={\text{c}}_{\text{gs}}(\text{a})\frac{\sum_{\text{r}}\int_{\alpha_{1}}^{\alpha_{2}}{\text{c}}_{\text{hr}}(\alpha )[\beta_{1}{\text{Y}}_{\text{hr}}(\text{t},\text{a})+\beta_{2}{\text{C}}_{\text{hr}}(\text{t},\text{a})]\text{d}\alpha }{\sum_{\text{r}}\int_{\alpha_{1}}^{\alpha_{2}}{\text{c}}_{\text{hr}}(\alpha ){\text{N}}_{\text{hr}}(\text{t},\alpha )\text{d}\alpha }$ Horizontal transmission coefficient: estimated from reported acute infections. β_1_: heterosexual: 0.33, homosexuals: 0.46 β_2_: heterosexual: 0.25, homosexuals: 0.30 0.013 for 0⩽a<15, 0 for a⩾15	p(a) = exp(−0.645a^0.455^)	8.667 per year	3.467 per year	0.015 per year	Acute: 0.724; Carrier: 0.115
Pang, et al (2010)^18^	Time-dependent. λ = β(y + αc), β: 0.85, estimated from the reported acute hepatitis B data by Ministry of Health of China.	0.1	6 per year	4 per year	0.005–0.025 per year	Carrier: 0.7–0.9
O’Leary, et al (2010)^23^	Time-dependent. λ = β(y(t) + αc(t)), α = 0.5, β: decided by basic reproduction number	p(λ) = f + (1 − f)exp[−0.645λ^−0.455^] Where f = 0.05	6 per year	4 per year	0.025 per year	Carrier: 0.11
Zou, et al (2010)^19^	Time-dependent, λ = β(y + αc) β: 1, estimated from the reported acute hepatitis B data by Ministry of Health of China	0.885	6 per year	4 per year	0.025 per year	Carrier: 0.11
Zou, et al (2010)^20^	Age-time-dependent, following Zhao, et al (2000)	p(a) = 0.176501 exp(−0.787711a) + 0.02116	6 per year	4 per year	0.025 per year	Carrier: 0
Mann, et al (2011)^26^	Age-time-dependent. $\lambda_{\text{j}}=\frac{\beta_{\text{j}}}{\sum_{\text{k}=1}^{5}{\text{N}}_{\text{k}}}\sum_{\text{i}-1}^{5}{\text{m}}_{\text{ij}}({\text{I}}_{\text{i}}+\alpha {\text{C}}_{\text{i}})$ β: 39.3, α: 0.05, m_ij_ describing the mixing pattern between age class i and j, the value is assumed.	p(a) = exp(−0.645a^0.455^)	1/0.1-transition rate	1/0.12 per year	0.03 per year	Carrier: 0.11
Zhang, et al (2012)^21^	Time-dependent. λ = β(y + αc), determined by the least square method comparing the model simulation and the demographic data and epidemiological data, α = 0.1, β = 1.1387	0.885	6 per year	4 per year	0.025 per year	Carrier: 0.11
Kamyad, et al (2014)^28^	Time-dependent. λ = β(y + αc), following Edmunds, et al (1996) β: 0.8–20.49	0.05–0.9	6 per year	4 per year	0.025 per year	Carrier: 0.11
Liang, et al (2015)^22^	Age-time-dependent, estimated from serological data using polynomial catalytic infection model λ(a,0) = 0.013662 + 0.280112 exp(−0.651583a)	<1 year, 0.3 1–5 years, 0.25 6–19 years, 0.06 ⩾20> years, 0.04	—	—	0.01 per year	Ignore intrauterine infection, the intrapartum and postpartum infection were reflected in the force of HBV infection

#### Force of infection and transmission coefficients

The force of infection (λ) is defined as the probability per unit of time that a susceptible individual becomes an infected individual. According the formula of the force of infection^29^ $\lambda =\text{k}\beta \frac{\text{C}}{\text{N}}$, this probability relies on the transmission coefficient (κβ) and the proportion of infectious individuals (C/N) in the population. The transmission coefficient represents the rate of effective contacts and the probability of transmission between individuals within a population. In the published studies, the force of HBV infection was directly estimated from serological data using the catalytic infection model (*n* = 4) or indirectly calculated from the transmission coefficient and the proportion of infectious individuals in the population (*n* = 9). Transmission coefficients were estimated from reported acute hepatitis B data (*n* = 4) or determined by basic reproduction number (*n* = 1). The others were assumed or followed an assumption from a previous paper. Given the age- or time-dependent factor, the force of the HBV infection was taken as age- and time-dependent (*n* = 7) or only time-dependent (*n* = 7). In terms of age factor, the continuous (*n* = 5) or discrete (*n* = 2) age distribution were applied to simulating age differences in the force of the HBV infection. In terms of time factor, the force of the HBV infection changing over time is only dependent on the proportion of infectious individuals in all of the recruited studies, yet the transmission coefficient remains the constant.

#### Proportion of acute infected individuals that become chronic carriers

The proportion of acute infected individuals that become chronic carriers was defined as age-dependent (*n* = 5), force of infection-dependent (*n* = 3), or constant (*n* = 5).

#### The transition rates from “latent”, “acute”, and “carrier” compartments

As the average time the infected individuals spend in the latent period is usually between 1.5 to 2 months, and the time spent in the acute period is 3 to 4 months, the rate at which individuals leave the latent class was defined as between 6 to 8 per year. The rate at which individuals leave the acute class was defined as 3 to 4 per year. The recovery rate of carriers was defined as age-dependent (*n* = 1) or constant (*n* = 12), which resulted in a value of 0.005 to 0.03 per year.

#### Proportion of perinatal infections

Most of the published studies ignored perinatal HBV infection caused by acutely infected mothers (*n* = 10). Others found this proportion to be about 0.7 (*n* = 3). The proportion of perinatal infections in newborns born from HBV carrier mothers was ignored in three studies. Ten studies found this proportion to be about 0.11, and one study found it to be 0.7–0.9. Two studies ignored intrauterine infection, and the intrapartum and postpartum infection with HBV were reflected in the force of HBV infection.

### The impact of vaccination

Based on the age-dependent mathematical models of HBV transmission (Figure 2) and the previously described parameter values (Table 1), these papers explored the impact of different vaccination strategies on the hepatitis B infection in different areas. As summarized in Table 2, the forecast period of the modeling was set to 14–150 years. The evaluated vaccination strategies were newborn vaccination, targeted vaccination, and vaccination of susceptible individuals. The simulation results suggest that the mathematical models provide a useful framework for evaluating the impact of vaccination strategies. In high HBV endemic areas, the vaccination strategies in newborns played the most important role in reducing HBV prevalence, and immunization of susceptible adults or highly risk groups or screening for chronic hepatitis B have a moderate additional effect on controlling HBV infection. However, in low HBV endemic areas, immunization of newborns has relatively poor effect in reducing HBV prevalence, while vaccination of newborns after antenatal screening or targeted vaccination program is more effective.

**Table 2.** The impact of vaccination strategies on HBV transmission

Endemicity (HBV carrier rate)	Example area	Predictive period	Infection control strategies	Conclusions	Reference Authors (year)
High (7–20%)	Gambia	100 years	Newborns	Numerical simulations of the model are shown to capture the observed age-specific patterns of serological markers. The eradication of HBV may be achieved by immunizing less than 70 per cent infants.	Edmunds (1996)^15^
	China	50 years	Newborns	The simulation results match the HBV epidemic data in China approximately. The immunization at birth should be improved as much as possible to decrease the HBsAg prevalence.	Zhang (2012)^16^
	China	50 years	Newborns, susceptible adults	Numerical simulations are performed to find optimal strategies for controlling the transmission of HBV. The optimal control strategy is a combination of immunization of newborns and retroactive immunization of susceptible adults.	Zou (2010)^20^
	China	14 years	Newborns	Newborn vaccination could account for more than 50% of the reduction of the total HBV prevalence. For the 2005 birth cohort which had high levels in the two coverage rates, the contribution rate could reach more than 95%.	Liang (2015)^22^
Low (<2%)	United Kingdom	50 years	Newborns and targeted	The model provides a useful framework for evaluating costs and benefits of immunization programs. Screening before vaccination markedly increases payback per dose in homosexuals but not in heterosexuals; infant vaccination gives the poorest effectiveness ratio and vaccination of infants after antenatal screening the best.	Williams (1996)^24^
	Netherlands	50 years	Targeted	Vaccination of children of immigrants from high and medium endemic countries is an effective strategy in countries with substantial immigration of carriers from high and medium endemic countries. A targeted vaccination program for sexually highly active risk groups has a moderate additional effect if continued over a long time period.	Kretaschmar (2009)^25^
	New Zealand	150 years	Newborns	The model captures aspects of the epidemic and highlights the areas where data and knowledge of parameter values are present, and could be built upon to model the effect of targeted vaccination campaigns. The vaccination campaign will decrease the number of carriers, but the carriers will continue to provide a route of infection to those still susceptible.	Mann (2011)^26^

## DISCUSSION

The spread of hepatitis B is a very complicated process. It is restricted and influenced by many interacting factors, including the numbers of susceptible and infectious individuals, the virulence and genotype of HBV, the length of latency period, the total population size, and the presence of medical interventions. To accurately predict the prevalence of hepatitis B and successfully apply predictions to the design of optimal, feasible public health policy for HBV prevention and control, one must construct a reasonable and attainable mathematical model. In this type of model, a variety of factors must be considered, including the population characteristics, natural history, and transmission pattern of hepatitis B.

The compartmental model is an effective tool to assess theoretical and practical contributions affecting the transmission of infectious disease.^30^^,^^31^ The remarkable features of these models are that the populations were stratified into different compartments according to the infectious states of disease. In the published studies, five- or six-compartment models are the most common model structure, the former including “Susceptible”, “Latent”, “Acute”, “Carrier”, and “Immune” and the latter further partitioning the “Immune” category into “Recovery” and “Vaccinated”. This is due to the fact that immunity after HBV recovery lasts the lifespan of the individual, while immunity following vaccination may wane over time. Especially, Liang et al^22^ developed the three-compartment model, only including the “susceptible”, “immune or vaccination”, and “chronic infection” compartments, to assess the independent impact of newborn vaccination. This model is a new view in compartmental models of HBV transmission. Although this simplified model can not reveal the whole natural process of HBV infection, it has advantages in the acquisition of the initial value of each compartment via epidemic survey data. Meanwhile, in comparison with the long-term natural history of chronic hepatitis B, the acute and latent infection with HBV is transient. So, this simplified model is feasible.

Based on the constructed epidemiological compartments, mathematical equations were used to describe the dynamic process of HBV transmission. Most of the published studies used differential equation models. In these models, ordinary differential equations or partial differential equations were constructed. Only two studies proposed a discrete, difference equation model with age structure. Due to the advanced theories of differential equations and dynamical systems, the differential equation model has been widely applied and well developed in modeling the transmission dynamics of HBV. However, the time unit of collection of data about epidemic transmission is usually weeks, months, or years, so it is more natural and convenient to construct a difference equation model with age structure, and we can easily compare the model result with the real data. Moreover, parameter estimation for discrete age-structure models can be, in general, relatively easy to compute. Many discrete age-structure models may also exhibit richer dynamical behaviors, even though it may be more challenging in theoretical study.^32^ Based on these features, a difference equation model with age structure is recommended for use in the prediction of hepatitis B prevalence.

The compartmental model is also associated with population characteristics. The complex process of HBV transmission may be influenced by variable population size and age structure. Most of the reviewed studies assumed constant population size, which neglected to take into account the changing population structure. This may affect the long-term forecast results. Age-dependence is one of the most important characteristics in the transmission of HBV, since the force of the HBV infection and the risk of HBV chronicity are related to age.^33^^–^^35^ Meanwhile, individuals with different ages may have different survival rates. To study the transmission dynamics of HBV and evaluate the effectiveness of mass vaccination programs, especially in newborns, the two important factors described above may have crucial influence and must be considered. These factors may affect the optimal timing of immunization schedules.^36^ Unfortunately, in the published studies, only 7 studies developed an age-structure model by considering an age-dependent force of infection. In addition, only 5 studies set an age-dependent probability of developing to a carrier state. So, in future studies, an age-structure model with variable population size and related age-dependent parameters should be considered when modeling.

The force of infection, which provides the rate at which newly infected individuals emerge, is an important epidemiological parameter in compartmental models.^37^ The force of HBV infection is the age-dependent parameter, because the contact patterns of HBV from the susceptible to the infected class in each age group are different. Meanwhile, it is changing over time, as the consequence of changes in the virulence of the pathogen during treatment and other health interventions or the contact patterns in each age group.^38^^,^^39^ Generally, the age-dependent force of infection can be directly deduced from a single cross-sectional serological survey, where data regarding individuals at different ages are analyzed using the catalysis model, while assuming infectious disease in the steady state.^40^ However, with the introduction of vaccination and other health interventions, this stationary situation is untenable and unrealistic. So, time-serial seroprevalence data should be used to estimate the change in the force of infection over time using a fully parametric or semi-parametric model.^41^^–^^45^ The force of infection can also be estimated using the incidence data reported through the national epidemic surveillance system; however, the incidence data of hepatitis B were only the symptomatic clinical cases. In fact, only 1% of neonates, 10% of children aged 1–5 years old, and a third of adults with HBV infections are symptomatic, and most of cases infected with HBV in infancy and early childhood did not experience clinical symptom until adulthood. That means it is not suitable to apply the incidence data in estimating the force of HBV infection. In the recruited studies, only 7 studies take into account the differences in the force of hepatitis B infection in each age group. All of the studies take into account the changes in this parameter over time, but time-dependent force of HBV infection only changed with the proportion of infectious individuals, but without considering the change of transmission coefficient over time. So, bias in the force of HBV infection and transmission coefficient will cause predictions to deviate from the true prevalence of hepatitis B.^37^

Vertical transmission, including the intrauterine, intrapartum, and postpartum transmission, is an important route of HBV transmission, especially in high prevalence areas. In the published papers of HBV transmission dynamics model, most scholars did not distinguish between intrapartum and postpartum intrauterine infection of HBV, only generally estimating the proportion of newborns with perinatal infection born to acute or carrier mothers. Those newborns with perinatal infection entered the latent or carrier compartment. In contrast, in the studies of Zhao et al^16^ and Liang et al,^22^ intrauterine infection was ignored and intrapartum and postpartum infection with HBV were reflected in the force of HBV infection; each newborn was considered susceptible to HBV at birth. At present, the intrapartum and postpartum infection with HBV can be blocked by the hepatitis B vaccine and immune globulin, but there is no effective method to prevent intrauterine transmission. In the modeling of HBV transmission, ignoring intrauterine HBV infection might result in a bias; however, this bias should not be large due to the very low incidence of intrauterine infection.^16^

In addition to age and transmission route, the accumulated evidence indicates that HBV genotype is closely related to the clinical outcome of hepatitis B.^46^^–^^49^ HBV genotype distribution has certain regional and ethnic characteristics, so the dynamics of the epidemic of HBV infection may differ according to the high-frequency genotypes in the difference area. However, there is no published paper on mathematical modeling of HBV infection considering HBV genotype. These may affect modeling results.

Based upon the constructed model structure and related values of parameters, the recruited studies made realistic simulations and then quantitatively analyzed the impact of vaccination strategies, mainly including newborn vaccination and targeted vaccination. The results suggest that the compartmental models focused on vaccination strategies have proposed some useful strategies for controlling the transmission of HBV.

In conclusion, so far, the dynamics model of HBV transmission is only from the macroscopic point of view to simulate the spread of HBV in the population. There are several main shortcomings in the model structure and parameter estimation from the modeling of these studies. First, age-dependence is the most important characteristic in the transmission of HBV, but an age-structure model and related age-dependent parameters were not adopted in some of the compartmental models describing HBV transmission. In addition, the numerical estimation of the force of HBV infection did not give sufficient weight to the age and time factors and is not suitable using the incidence data. Lastly, the current mathematical models did not well reflect the details of the factors of HBV transmission, such as migration from high or intermediate HBV endemic areas to low endemic areas and the kind of HBV genotype. All of these shortcomings may lead to unreliable results. When the mathematical model closely reflects the fact of hepatitis B spread, the results of the model fit will provide valuable information to controlling the transmission of hepatitis B.
